# A Picture is Worth a Thousand Timesteps: Excess Entropy Scaling for Rapid Estimation of Diffusion Coefficients in Molecular-Dynamics Simulations of Fluids

**DOI:** 10.1021/acs.jctc.4c00760

**Published:** 2024-11-07

**Authors:** S. Arman Ghaffarizadeh, Gerald J. Wang

**Affiliations:** † Department of Mechanical Engineering, Carnegie Mellon University, 5000 Forbes Avenue, Pittsburgh, Pennsylvania 15213, United States; ‡ Department of Civil and Environmental Engineering, Carnegie Mellon University, 5000 Forbes Avenue, Pittsburgh, Pennsylvania 15213, United States

## Abstract

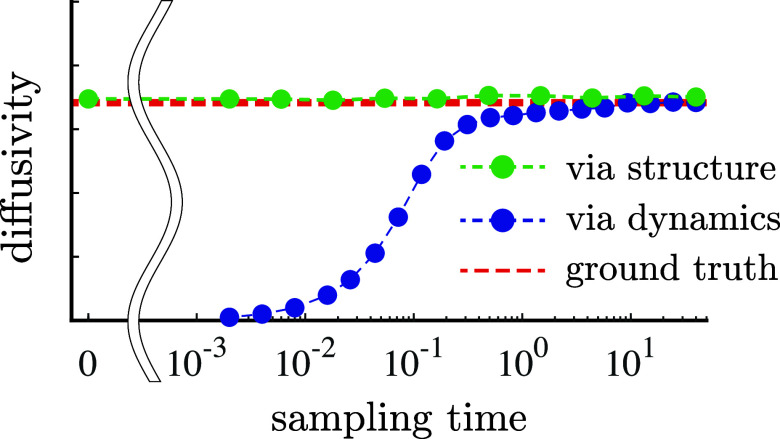

In molecular-dynamics simulations of fluids, the Einstein–Helfand (EH) and Green–Kubo (GK) relationships are frequently used to compute a variety of transport coefficients, including diffusion coefficients. These relationships are formally valid in the limit of infinite sampling time: The error in the estimate of a transport coefficient (relative to an infinitely long simulation) asymptotically approaches zero as more dynamics are simulated and recorded. In practice, of course, one can only simulate a finite number of particles for a finite amount of time. In this work, we show that in this pre-asymptotic regime, an approach for estimating diffusion coefficients based upon excess entropy scaling (EES) achieves a significantly lower error than either EH or GK relationships at fixed online sampling time. This approach requires access only to structural information at the level of the radial distribution function (RDF). We further demonstrate that the use of a recently developed RDF mollification scheme significantly reduces the amount of sampling time needed to converge to the long-time value of the diffusion coefficient. We also demonstrate favorable sample-to-sample variances in the diffusion coefficient estimate obtained using EES as compared to those obtained using EH and GK.

## 1 Introduction

In any system where the thermal energy-scale is at least comparable to the energy-scale of interatomic interactions, a transport quantity of interest is the mass diffusivity. In molecular-dynamics (MD) simulations of fluids, there are numerous techniques for measuring self-diffusion coefficients,^1,2^ two of which are by far the most popular: the Einstein–Helfand (EH) approach and the Green–Kubo (GK) approach.^3,4^ Both methods require data to be collected on an otherwise-equilibrated system over a window of time *t*_sample_; ideally, to reduce statistical errors (and modulo computational limitations), data is collected in the asymptotic limit *t*_sample_ → ∞. In this work, we explore a naturally complementary question: Given a fixed, finite *t*_sample_, what method enables the highest-fidelity estimate of the true self-diffusion coefficient? Questions of this nature—“how well can we estimate transport quantities in the pre-asymptotic regime?”—take on particular importance in an age of high-throughput materials simulation, optimization, and reinforcement learning: Even if a single self-diffusion coefficient is relatively inexpensive to obtain, a high-throughput screening effort can easily demand orders of magnitude more data points. Such workflows demand large volumes of simulation data (as is the case, e.g., for the population of a materials property database) and/or quick determinations that a particular material or set of thermodynamic conditions is unfavorable, so that the next candidate material or set of conditions can be evaluated (as is the case for, e.g., a device-optimization routine). In all of these cases, faster (even if potentially lower-accuracy) estimates can play a critical role.

In this work, we compare three approaches for computing the self-diffusion coefficient: the two mentioned above (EH and GK) and excess entropy scaling (EES). This last approach, which we describe in detail below, has been successfully used to inform models for diffusion in a variety of materials, including hard-sphere fluids,^5^ Lennard-Jones (LJ) fluids,^6,7^ supercooled and binary mixtures,^8−10^ ionic melts,^11^ hydrocarbons,^12^ a wide diversity of coarse-grained models,^13−16^ and active materials.^17−20^ The core potential benefit of this third approach is that it can circumvent pre-asymptotic limitations inherent to the other two methods, as it relies upon only configurational entropy, which can be computed using only positions (and not momenta) drawn from equilibrium. To date, no work has compared these three methods in the context of pre-asymptotic estimates for self-diffusion coefficients across a wide range of thermodynamic conditions. The closest existing studies investigate time-dependent diffusion coefficients measured via EH and GK. Of particular note are studies by Chitra and Yashonath and by Zúñiga and Español on the effect of measurement window on measured diffusivity for EH^21^ and GK;^22^ by Kim et al.,^23^ which investigates time-dependent diffusion coefficients obtained from the Langevin equation under a variety of noise models and from MD simulations of simple fluids at a single temperature and density; and by Heyes et al.,^24^ which models time-dependent transport coefficients by integrating quasi-empirical forms^25,26^ for the associated time-correlation functions (which are in turn parametrized via extensive fitting to MD simulations).

## 2 Methodology

### 2.1 MD Simulations

To investigate the effect of sampling window on measurements of the self-diffusion coefficient in a fluid system, we perform three-dimensional MD simulations of *N* = 1500 fluid particles with mass *m* placed in a cubic domain with number density ρ (with periodic boundary conditions applied in all three dimensions) interacting via the LJ potential^3,4^
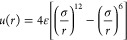
1where *u*(*r*) is the interaction energy of two particles separated by a distance *r*, and ε and σ are energy- and length-scales for the LJ potential, respectively. In all discussions that follow, unless otherwise stated, all quantities are nondimensionalized against the length-scale σ, energy-scale ε, mass-scale *m*, time-scale , density-scale *m*/σ^3^, diffusivity-scale , entropy-scale *k*_B_, and temperature-scale *k*_B_/ε, where *k*_B_ is the Boltzmann constant. Simulations are conducted using the large-scale atomic/molecular massively parallel simulator^27^ (LAMMPS) with a timestep of 2 × 10^–3^. A box side length is selected in the range 11.45 ≤ *L*_box_ ≤ 12.89, corresponding to number densities in the range 0.7 ≤ ρ ≤ 1.0. Using the Nosé-Hoover thermostat,^28,29^ each simulation is run in the canonical ensemble for a time of 50 to attain a specific target temperature in the range 0.8 ≤ *T* ≤ 2. The system is then switched to the microcanonical ensemble and allowed to equilibrate for an additional time of 50. Once equilibrated, the system is run for *t*_sample_ = 40 for sampling purposes, with kinematic data recorded every timestep. To obtain representative statistics, 1000 independent simulations were performed, featuring in each case variations in both the initial particle position and the initial assignment of particle velocities (samples are drawn from a Maxwell–Boltzmann distribution at the target temperature for the system under study).

### 2.2 Measurement Techniques for the Self-Diffusion Coefficient

We use three methods to measure the coefficient of self-diffusion *D*.

#### 2.2.1 Einstein–Helfand

**Figure 1:**
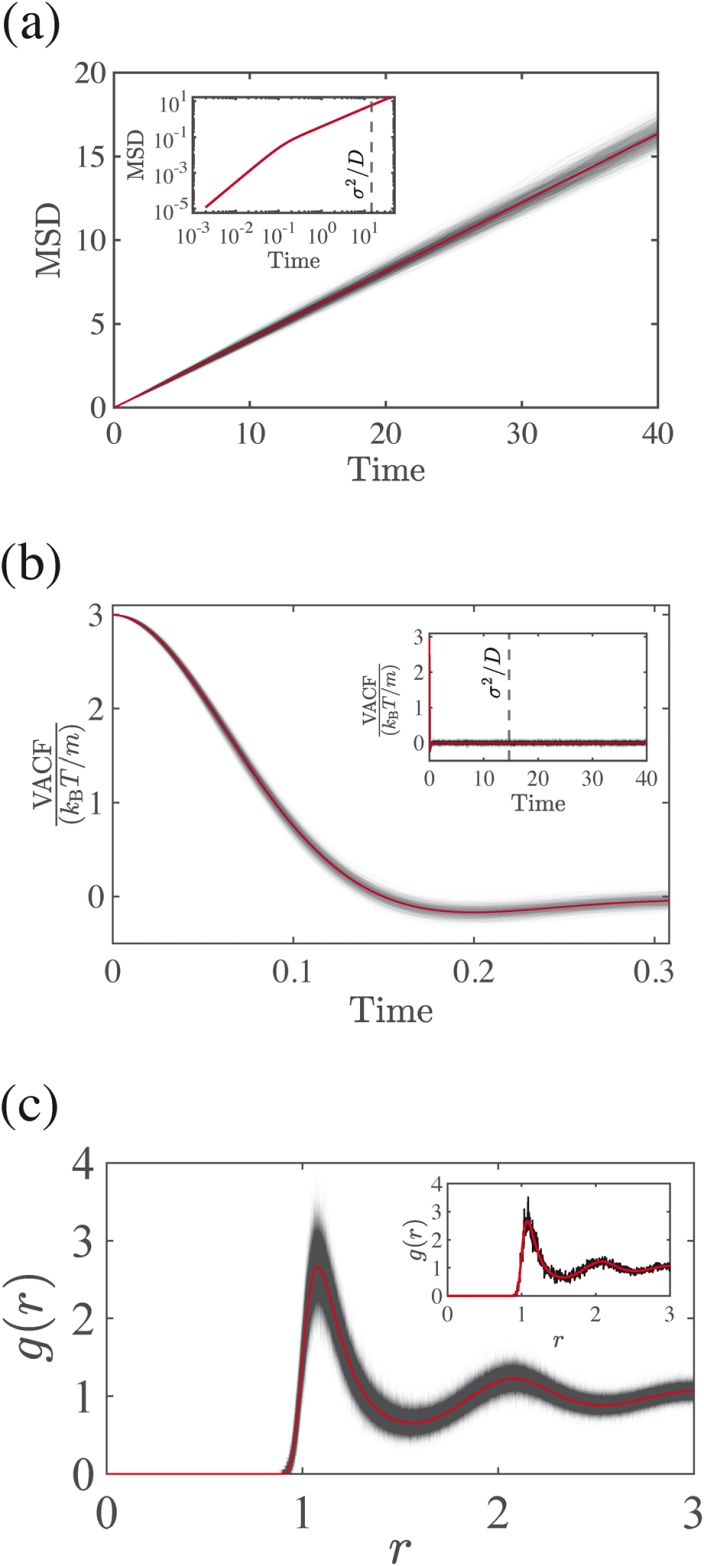
Three approaches for measuring self-diffusion coefficients: (a) EH, which makes use of MSD vs time data. Inset shows the early-time behavior of MSD, exhibiting a ballistic regime in which MSD scales as time squared; the ballistic regime is clearly finished by the (dimensional) diffusional time-scale σ^2^/*D*. (b) GK, which makes use of VACF vs time data. Inset shows the long-time behavior of the VACF, which tends toward zero in ensemble average. (c) EES, which makes use of the excess entropy, which can be approximated using the RDF *g*(*r*). Inset shows an instantaneous RDF from a single timestep. Throughout, results are shown for 1000 statistically independent runs (black), with the average overlaid (red).

We measure the mean-squared displacement (MSD) as *S*(*t*) ≡ ⟨||**r**^2^(*t*)||⟩ = ⟨||**r**(*t*) – **r**(0)||^2^⟩, where **r** is the position vector of a reference particle, *t* denotes the time at which the measurement is taken, and the angle brackets indicate an average computed over all particles serving as the reference particle. *D*_EH_ is obtained via least-squares by regressing *S*(*t*) against the straight-line model 6*D*_EH_*t*. Figure 1a shows example MSD profiles.

#### 2.2.2 Green–Kubo

We compute the velocity autocorrelation function (VACF) as *Z*(*t*) = ⟨**v**(*t*)·**v**(0)⟩, where **v** is the velocity vector of a reference particle and, again, the angle brackets indicate an average computed over all particles serving as the reference particle. *D*_GK_ is obtained as
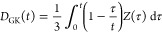
2but is often approximated as
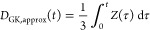
3for the reason that this expression is evaluated in the long-*t* limit (see Appendix A for a discussion of the relationship between *D*_GK_ and *D*_EH_). Figure 1b shows example VACF profiles.

#### 2.2.3 Excess Entropy Scaling

We approximate the excess entropy *S*_exc_, which is defined as the difference between a system’s entropy and the ideal gas entropy at the same temperature and density. Note that this quantity is necessarily negative, as the ideal gas has the highest possible entropy at any fixed temperature and density. Throughout this work, we approximate the excess entropy using the two-particle-correlation approximation^30−34^
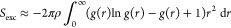
4where *g*(*r*) is the radial distribution function (RDF), which can be obtained at any given timestep and also averaged over multiple timesteps. Figure 1c shows example RDF profiles. In practice, the integral in eq 4 is numerically evaluated to a maximum radius *r*_max_. Throughout this work, we employ *r*_max_ = 4; the negligible effect of this finite-radius cutoff is discussed in Appendix B. For the simple fluid at the temperatures and densities studied in this work, the two-particle-correlation approximation represents a significant majority of the total excess entropy.^35−37^

*D*_EES_ is obtained as
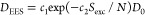
5where *D*_EES_ is the fluid’s coefficient of self-diffusion, and *c*_1_ and *c*_2_ are positive and material-specific constants that are obtained from prior (and off-line) regression of *D* (measured using either EH or GK) against the exponential model . In dimensional terms, the baseline diffusivity .^38^ Details on the convergence of *c*_1_ and *c*_2_ with respect to number of training points is presented in Appendix C. For the LJ systems studied herein, we use *c*_1_ = 0.45 and *c*_2_ = 0.75.

To improve the robustness of the EES approach when using eq 4, we also study the effect of using the Kernel-Averaging Method to Eliminate Length-of-Bin Effects (KAMEL-LOBE). In brief, the idea behind KAMEL-LOBE is to systematically mollify each RDF so that the (arbitrary) choice of RDF histogram bin width (which we denote as Δ*r*) has negligible effect on the excess entropy estimated using eq 4. The method is outlined in Appendix D. Full details on the theoretical basis and numerical implementation of this technique, an analysis of the impact of RDF histogram bin width on the EES approach, and a review of other methods for systematic mollification of RDFs can be found in recent work by the authors.^39^

## 3 Results and Discussion

**Figure 2:**
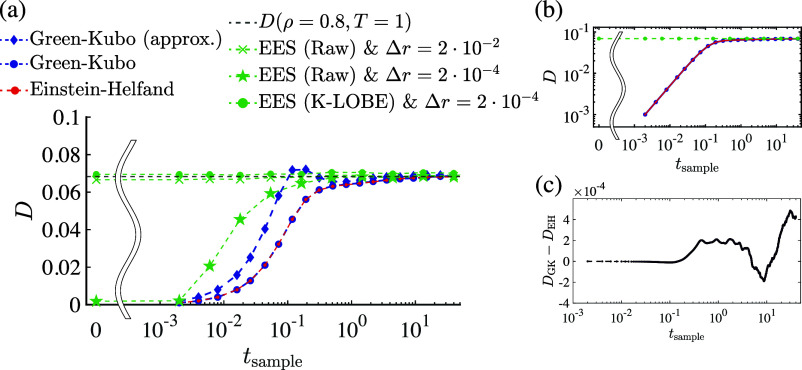
On (a) linear and (b) logarithmic axes, self-diffusion coefficient for a system with ρ = 0.8 and *T* = 1 as a function of sampling time for the exact GK (eq 2, blue circles), the approximate GK (eq 3, blue diamonds), EH (red circles), and EES, using both unprocessed (raw) RDFs (green x’s and stars) and RDFs processed using KAMEL-LOBE (green circles). At each value of *t*_sample_, GK and EH results are averages over 1000 statistically independent replicates; EES results are from a single replicate. Dashed lines are provided as a guide to the eye. The black dashed line corresponds to the self-diffusion coefficient at these thermodynamic conditions reported by Meier et al.^40^ (c) Discrepancy between self-diffusion coefficients obtained using EH and using exact GK (eq 2), which is on the order of magnitude of numerical integration error.

Figure 2 illustrates the self-diffusion coefficient for EH, GK, and EES methods as a function of sampling time for a system with ρ = 0.8 and *T* = 1, compared against the self-diffusion coefficient reported by Meier et al.^40^ In the pre-asymptotic regime, both EH and GK underestimate the long-time value, a trend consistent with results in refs (22, 23, 41–45). In particular, for *t*_sample_ ≲ 10^–1^, both the EH and GK methods underestimate the long-time value by at least 40%; the error does not fall to ∼1% until the diffusional time-scale *D*^–1^. In the case of EH, the slope of the best-fit line in the ballistic regime (where MSD is quadratic in time) falls below the slope of the best-fit line in the diffusive regime, and this effect persists even beyond the transition time-scale. It is of course not to be expected that a straight-line model would accurately capture the MSD-time relationship in the short-time regime, which highlights an intrinsic limitation in the use of EH for rapid estimation of diffusion coefficients (in particular, on time-scales less than ). In the case of GK, which contains the same information as EH, the underestimate occurs because there still remains a significant amount of VACF yet to be integrated. With regard to GK in the pre-asymptotic regime, Figure 2a also highlights the importance of the (1 – τ/*t*) factor in the integrand (which is frequently discarded on the basis that it approaches unity in the infinite-time limit) for the purposes of obtaining agreement between GK and EH (Figure 2c). Ironically, since the exact GK relation leads to systematic underestimates at short sampling times and since (1 – τ/*t*) is bounded from above by unity for 0 ≤ τ ≤ *t*, the approximation in eq 3 actually leads to lower errors throughout the pre-asymptotic regime. This serendipitous cancellation of errors—an underestimate due to premature truncation of a largely positive integrand and an overestimate due to dropping a negatively signed term from integration by parts—can generally be expected, which points to an underappreciated (if also inadvertent) merit of the approximation in eq 3.

In contrast, EES predicts a diffusion coefficient at early times that is much closer to the long-term predictions of both ER and GK, provided that the bin width Δ*r* is judiciously selected (e.g., in Figure 2, Δ*r* = 2 × 10^–2^). In particular, using only a single snapshot, which requires no dynamics (beyond that required to reach equilibration, i.e., *t*_sample_ = 0), an estimate for *D* can be obtained that is within 4% of its long-time value, with lower-magnitude errors as *t*_sample_ increases.

**Figure 3:**
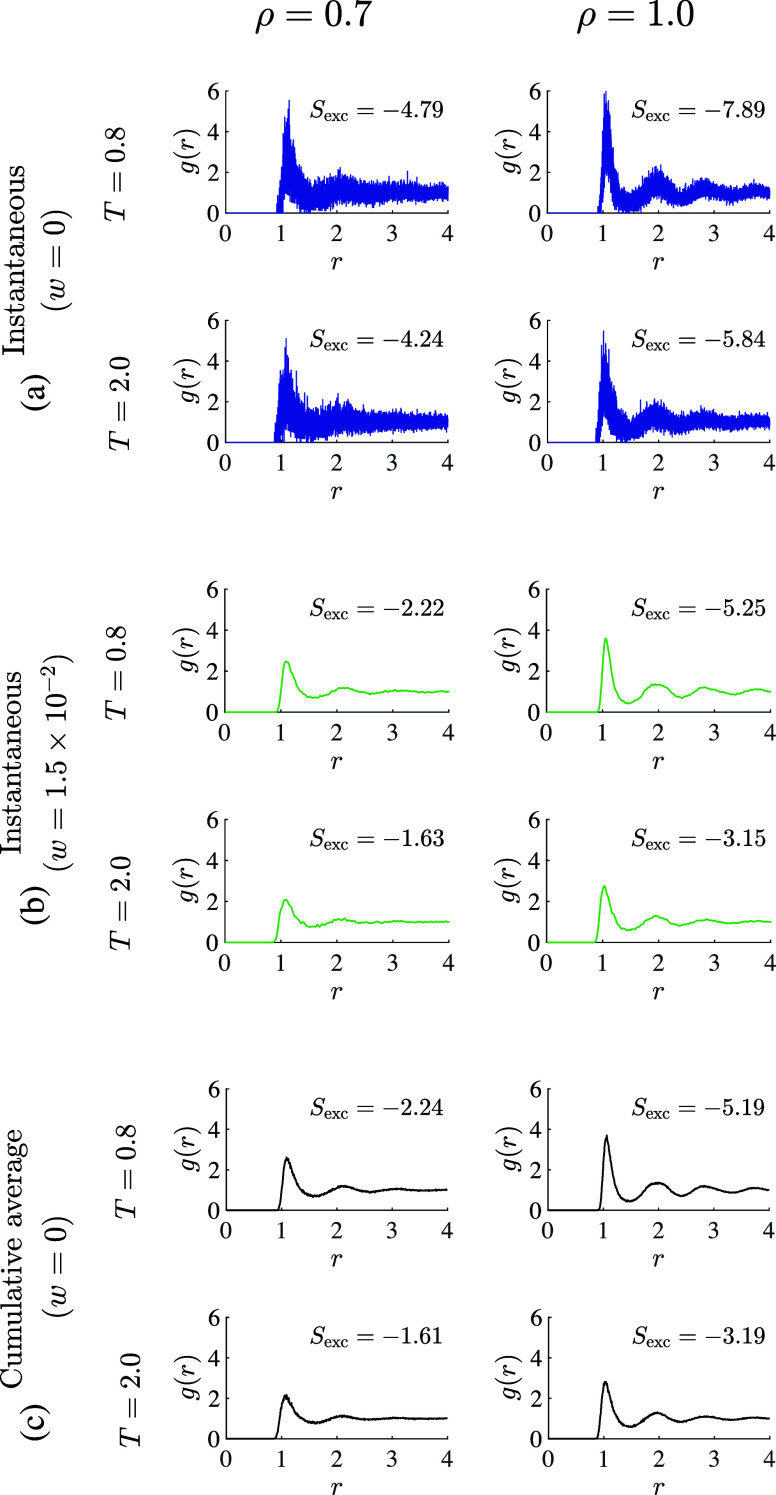
RDF profiles computed using Δ*r* = 4 × 10^–4^ at the maximum and minimum densities and temperatures studied herein, along with the excess entropy estimate from each profile computed using eq 4, for (a) a single equilibrium snapshot (*t*_sample_ = 0) with no use of KAMEL-LOBE (*w* = 0), (b) a single equilibrium snapshot processed using KAMEL-LOBE (*w* = 1.5 × 10^–2^), and (c) a long-time average making use of 11 RDF profiles equally spaced in time over *t*_sample_ = 40, with no use of KAMEL-LOBE.

If however the RDF is “overbinned” (that is, the bin width is chosen to be overly fine, e.g., in Figure 2, Δ*r* = 2 × 10^–4^, or in Figure 3, Δ*r* = 4 × 10^–4^), then *D*_EES_ will be an underestimate of the true value at early times (using a small number of snapshots), with error magnitudes that are on the order of those using EH and GK. As with all methods, this underestimate is ameliorated by progressively averaging *g*(*r*) over more snapshots, eventually converging to the same result as for the coarser choice of Δ*r*. It is worth noting that this underestimate at early times is systematic, for reasons discussed in Appendix E.

**Figure 4:**
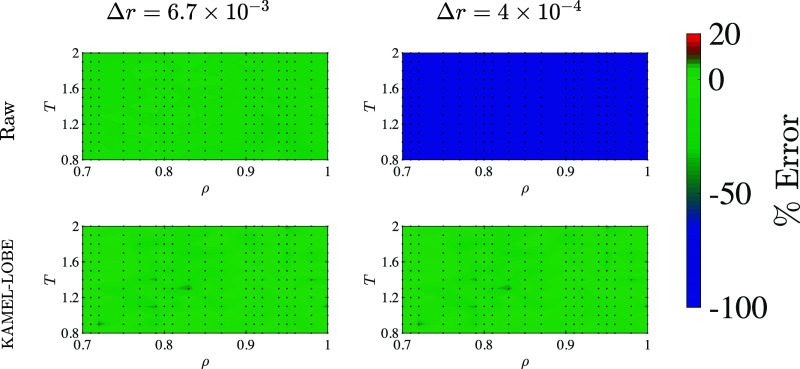
Percentage errors for *D*_EES_ as compared to long-time estimates obtained with a single snapshot, using two choices of bin width, and using raw RDFs and RDFs processed using KAMEL-LOBE (*w* = 1.5 × 10^–2^).

We find that the application of KAMEL-LOBE to an overbinned RDF can recover the long-time RDF profile, the long-time value of *S*_exc_, and thus the long-time value of *D*, even from a single snapshot (Figure 3). In Figure 2, *D*_EES_ with the use of KAMEL-LOBE is consistently within 2% of the long-time value, even from the first snapshot. In Figure 4, this result is extended to the full range of densities and temperatures studied. Whereas there are significant errors across all values of density and temperature in the overbinned case when using a single snapshot, these errors are reduced by nearly 2 orders of magnitude through the use of KAMEL-LOBE. Overall, these results point to the utility of mollification techniques to enhance entropy-scaling-based estimates of transport coefficients in the pre-asymptotic regime, unexplored in earlier work.^39^

**Figure 5:**
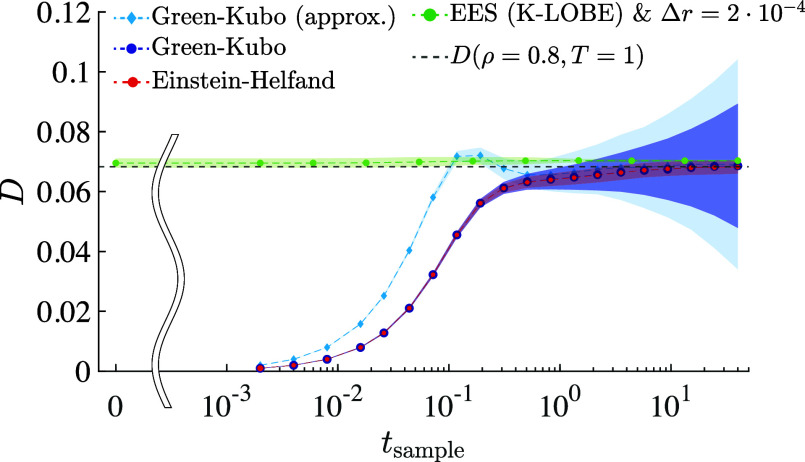
Self-diffusion coefficient for a system with ρ = 0.8 and *T* = 1 as a function of sampling time for the exact GK (eq 2, dark blue), the approximate GK (eq 3, light blue), EH (red), and EES with KAMEL-LOBE (green). At each value of *t*_sample_, shading for GK and EH represents a single standard deviation computed over 1000 statistically independent replicates; EES results are a single standard deviation computed over 100 statistically independent replicates. Dashed lines are provided as a guide to the eye. The black dashed line corresponds to the self-diffusion coefficient at these thermodynamic conditions reported by Meier et al.^40^

All of the results thus far concern the average value of the diffusivity inferred as a function of sampling time. It is also worth comparing sample-to-sample standard deviation across independent experiments performed using each method (Figure 5). In the regime of sampling time where all methods produce comparable results (*t*_sample_ ≳ 1), EES yields the lowest standard deviation among all methods compared, pointing to yet another benefit of this approach. As is well-known, the GK method produces a sample-to-sample standard deviation that diverges as the sampling time grows.^46,47^ Intriguingly, the approximate GK approach (which, as discussed earlier, yields a lower-error mean at early times) features the largest (and fastest growing in time) standard deviation of all approaches studied.

We conclude with a brief discussion as to why EES is capable of achieving a substantially lower-error estimate for the diffusion coefficient in the early time regime (at any fixed amount of online sampling time). In a system with *N* particles, at each timestep, EES leverages an amount of information that goes (roughly) as , whereas EH and GK leverage an amount of information that goes as . There is a statistical benefit to incorporating information about structure at the level of pair correlations when computing transport properties. As an additional benefit, because EES only uses structural information, it is amenable to pairing with molecular simulation techniques that do not explicitly perform dynamics (e.g., Monte Carlo simulation). It is of course also worth emphasizing that EES acts in an interpolative fashion in the space of excess entropies (and, by extension, in the space of possible particle configurations); any prediction made using EES leverages the fitting coefficients *c*_1_ and *c*_2_, which contain information about all off-line simulations (which must simulate dynamics) that are used to compute *c*_1_ and *c*_2_ in the first place.

## 4 Conclusion

In this work, we compare several methods for computing self-diffusion coefficients in molecular simulations: EH, GK, and EES, with a particular focus on the pre-asymptotic (early-sampling-time) regime. Whereas EH and GK make use of dynamical information, EES (at the level of approximation studied) uses only structural information contained within the RDF. We find that over a wide range of densities and temperatures, EES (when used in conjunction with a recently developed technique for RDF mollification) consistently yields the lowest error of all methods studied, even using just a single snapshot (no dynamics performed beyond equilibration). We also show that EES exhibits a small sample-to-sample standard deviation, as compared to the other methods, a difference that is especially pronounced at sampling times where all methods produce comparable results. Overall, these results point to the utility of EES as a technique for rapid estimation of transport coefficients in molecular simulations; in other words, in informal terms, when using EES, we find that a picture is (statistically) worth a thousand timesteps (and perhaps more).

The results presented herein raise a host of intriguing questions related to the use of EES for rapid estimation of transport coefficients, several of which will be the subjects of future work. For example, all of the present work presumes systems already at equilibrium; it is natural to wonder whether these same results might extend to systems out of equilibrium, whether due to active forcing or due to nonequilibrium transients. Another fruitful area of further inquiry pertains to the two-particle-correlation approximation in eq 4. Our work suggests that for simple fluids, computing the excess entropy at the level of the two-particle-correlation approximation is sufficient for the purposes of rapid estimation. This story is likely to be more complicated for systems where the approximation misses a considerable amount of the total excess entropy,^36,48^ given that the computational cost of approximating higher-body correlations in the first place may be incompatible with the goal of rapid estimation. It is worth noting, however, that the dominance of the two-particle-correlation term relative to higher-order terms is sufficient, but not necessary, for EES to work well as a rapid estimation scheme, since the fitting of the parameter *c*_2_ naturally absorbs some of the error introduced by the two-particle-correlation approximation. Although the present work focuses on bulk systems, another intriguing area for further study pertains to confined systems,^9^ especially in view of the potential challenge in structurally decorrelating such systems^49^ and in view of the diversity of approaches for quantifying diffusion under confinement in the first place.^2,50^ It may also be possible to connect these results, which demonstrate favorable variances for EES as compared to EH and GK, with recent work on a class of variance-reduced estimators for molecular simulation;^51−53^ a particularly intriguing outcome would be the development of a fused estimator merging these approaches.

## Figures and Tables

**Figure 6:**
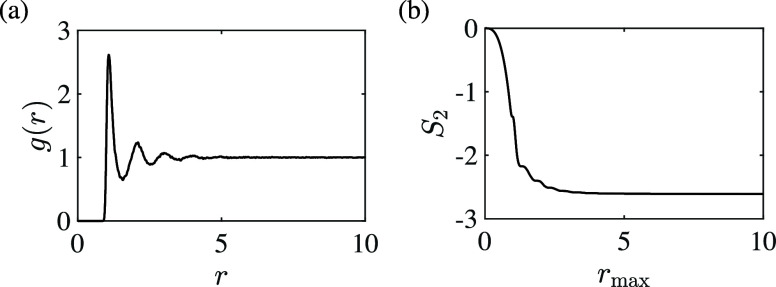
(a) RDF for a system with ρ = 0.8 and *T* = 1; (b) EES (two-particle-correlation approximation) as a function of the maximum radius considered. For both (a,b), KAMEL-LOBE has been employed.

**Figure 7:**
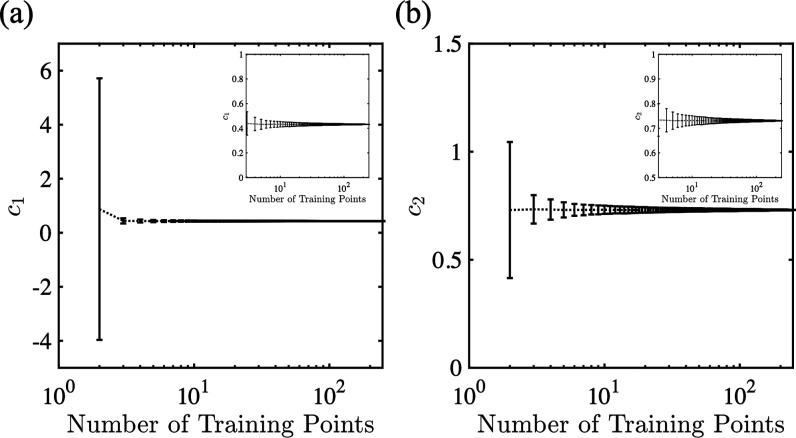
Dependence of (a) *c*_1_ and (b) *c*_2_ on the number of EH simulations over which each parameter is fit. Each EH simulation is run for a period of *t*_sample_ = 500. Error bars shows one standard deviation for the value of each parameter, evaluated over 1000 randomly drawn subsets of the appropriate size. Dashed lines connect the mean values of each parameter, evaluated over these 1000 subsets. To improve visibility of error bars, the insets show the same data, but restricted to three or more training points.

## Data Availability

An implementation of and tutorial notebook for KAMEL-LOBE is available at https://github.com/M5-Lab/KAMEL-LOBE.

## Appendix A

### 4.1 Relationship between *D*_EH_ and *D*_GK_

In this Appendix, we discuss the relationship between the EH and GK methods, starting with a derivation of the formal equivalence between the two methods and a brief comment on their (in)equivalence in typical MD practice.

We first show that eq 2 can be obtained directly from the EH approach, generally following the argument laid out by Hansen and McDonald.^54^ We start by recasting *S*(*t*) = ⟨||**r**(*t*) – **r**(0)||^2^⟩, which is a function of positions, into a function of velocities. To do this, we note that

6

7

8

The region of integration in the *t*′*t*″-plane is a square [with vertices that include the origin and (*t*, *t*)]. By symmetry, this integral is equivalent to twice the integral over the triangle with vertices at the origin, (*t*, 0), and (*t*, *t*)

9

Leveraging the definition of *Z*(*t*), this is the same as

10

Transforming the second integral via the change of variables τ ≡ *t*′ – *t*″, we obtain

11

Integrating by parts

12

13

The right-hand side of eq 13 is simply *D*_GK_ as expressed in eq 2; in the limit *t* → ∞, the quotient on the left-hand side of eq 13 gives the asymptotic value of *D*_EH_. As such, in the infinite-time limit, both methods are analytically equivalent (and, moreover, the τ/*t* term can be neglected, as discussed earlier).

However, it is worth emphasizing that computing the asymptotic and regressing *S*(*t*) against a straight-line model of the form 6*D*_EH_*t* at finite *t* to obtain the parameter *D*_EH_, while similar in spirit, are not identical mathematical operations. As such, for any given data set (collected over finite time), it is not necessarily true that *D*_EH_ and *D*_GK_ are exactly equal (and for a collection of such data sets, the distribution of diffusivities obtained via EH is not in general the same as the distribution obtained via GK).

## Appendix B

### 4.2 Convergence of Two-Particle-Correlation Approximation for Excess Entropy

In this Appendix, we study the effect of the finite upper bound *r*_max_ for the integral in eq 4; in other words, we are interested in

14

In Figure 6, we show that the first two peaks in *g*(*r*), which are separated by approximately unity, contribute the vast majority of *S*_2_. By *r*_max_ = 4 (used throughout this work), *S*_2_ is within 1% of its value at the largest cutoff studied (*r*_max_ = 10).

## Appendix C

### 4.3 Convergence of EES Parameters

In this Appendix, we explore the convergence of the two parameters *c*_1_ and *c*_2_ in eq 5 with respect to the number of EH simulations used to train the EES relationship. To do this, we generate a data set of 10^4^ EH simulations with *t*_sample_ = 500. We then select at random 1000 subsets of size *p* (the number of training points), where 2 ≤ *p* ≤ 250. For each subset, *c*_1_ and *c*_2_ are computed. In Figure 7, we observe as expected that as the number of training points increases, the average values of each parameter do not change (within statistical noise) but the standard deviation of each parameter decreases. These results inform the number of training points that should be used for EES, depending upon the precision required for the target application; as a rough rule of thumb, based upon these results, one should target *p* ≳ 10.

## Appendix D

### 4.4 KAMEL-LOBE Technique

In this Appendix, we describe in detail the KAMEL-LOBE technique.^39^ KAMEL-LOBE systematically mollifies RDFs in a mass-conserving fashion. Given a vector **g** with *n* values of the RDF at the radial coordinates , where  is the maximum radius considered, KAMEL-LOBE is a linear map that takes **g** to a smoothed **g̃** as , where

15

16

17

where  (*m*-by-*n*),  ((*n* – 2*m*)-by-*n*), and  (*m*-by-*n*) are

18

19

20

 denotes an *m*-by-*m* identity matrix, 0_*n*–*m*_ denotes an *m*-by-(*n* – *m*) matrix of zeroes, and the weight *u*_*j*_ is calculated as the area under the Gaussian probability density function within the *j*-th bin, which runs from *r* – (*k*/2 + *j* – 1)Δ*r* to *r* – (*k*/2 + *j*)Δ*r*. In other words, *u*_*j*_ = Φ[*r* – (*k*/2 + *j*)Δ*r*] – Φ[*r* – (*k*/2 + *j* – 1)Δ*r*], where Φ denotes the Gaussian cumulative distribution function. Smoothing is performed only for separation distances exceeding 2*w*. All results presented herein use *w* = 1.5 × 10^–2^, a broadly useful choice for *w* as established in earlier work.^39^

## Appendix E

### 4.5 Systematic Errors for EES in the Pre-Asymptotic Regime

In this Appendix, we sketch a proof that the errors for EES in the pre-asymptotic regime are systematic underestimates, as empirically shown in Figure 2. The general approach is to leverage the convexity of the entropy. We denote the asymptotic (infinite-time-average) RDF as *g*_∞_(*r*), for which the corresponding excess entropy (at the level of the two-particle-correlation approximation) is

21

For any amount of sampling time τ (and presuming a fixed frequency of RDF outputs), we denote the pre-asymptotic RDF obtained through averaging as *g*_τ_(*r*), which in general is not identical to *g*_∞_(*r*) for finite τ; as a consequence,  Since conservation of mass demands ρ∫_0_^∞^4π*r*^2^*g*_∞_(*r*) d*r* = ρ∫_0_^∞^4π*r*^2^*g*_τ_(*r*) d*r* = *N*, it is necessarily true that for any arbitrarily small radial separation ϵ, there exists *r*_0_ such that [*g*_τ_(*r*_0_ + ϵ) – *g*_∞_(*r*_0_ + ϵ)][*g*_τ_(*r*_0_) – *g*_∞_(*r*_0_)] < 0, i.e., *g*_τ_ is higher at one radius and lower at the other radius as compared to *g*_∞_ (in informal terms, the pre-asymptotic RDF is “more jagged” or “spikier” than the asymptotic RDF). Without loss of generality, let us focus on the excess entropy contribution from one localized patch of “spikiness” where *g*_τ_(*r*_0_) > *g*_∞_(*r*_0_) > *g*_∞_(*r*_0_ + ϵ) > *g*_τ_(*r*_0_ + ϵ), and so we can write

22

23

where δ_1_, δ_2_ > 0. [The same general argument applies if *g*_τ_(*r*_0_) < *g*_∞_(*r*_0_) < *g*_∞_(*r*_0_ + ϵ) < *g*_τ_(*r*_0_ + ϵ); the key point is that *g*_∞_(*r*) is smoother than *g*_τ_(*r*)].

The pre-asymptotic excess entropy integrand for these two radii is thus

24

which can be rearranged as

25

Assuming δ_1_ ≪ *g*_∞_(*r*_0_) and δ_2_ ≪ *g*_∞_(*r*_0_ + ϵ), we can expand eq 25 as

26

27

where we have omitted terms that are  or . Noting that the asymptotic excess entropy integrand for these two radii is

28

we find the difference between the pre-asymptotic and asymptotic integrands to be

29

This quantity is positive since we initially assumed *g*_∞_(*r*_0_) > *g*_∞_(*r*_0_ + ϵ). As a consequence, the excess entropy obtained using the pre-asymptotic RDF *S*_exc,τ_ is less than *S*_exc,∞_, and so by eq 5, the diffusivity inferred using the pre-asymptotic RDF is lower than that obtained using the asymptotic RDF.

A physical interpretation of this result is that the “spikier” pre-asymptotic RDF more closely resembles the RDF of a crystalline solid than the asymptotic RDF. As a consequence, transport coefficients obtained from the pre-asymptotic RDF will err toward crystalline-solid-phase values and away from ideal-gas values; in the case of diffusivity, the pre-asymptotic RDF yields an underestimate.
